# Assessment of heavy metal accumulation and health risk in three essential edible weeds grown on wastewater irrigated soil

**DOI:** 10.1038/s41598-023-48763-5

**Published:** 2023-12-08

**Authors:** Zinab A. Abdelgawad, Mona N. Abd El-Wahed, Asmaa A. Ahmed, Seliem M. Madbouly, Gharieb S. El-Sayyad, Ahmed A. Khalafallah

**Affiliations:** 1 Botany Department, Faculty of Women for Arts, Science and Education, Ain Shams University, Cairo, Egypt; 2 Chemistry Lab, Fresh Water Division, National Institute of Oceanography and Fisher (NIOF), Cairo, Egypt; 3 Microbiology and Immunology Department, Faculty of Pharmacy, Ahram Canadian University, Giza, Egypt; 4 Microbiology and Immunology Department, Faculty of Pharmacy, Galala University, New Galala City, Suez, Egypt; 5 Drug Microbiology Lab, Drug Radiation Research Department, National Center for Radiation Research and Technology (NCRRT), Egyptian Atomic Energy Authority (EAEA), Cairo, Egypt

**Keywords:** Plant sciences, Environmental sciences, Natural hazards, Planetary science

## Abstract

The main problem facing Egypt recently is the shortage of available water resources. Therefore, farmers resort to use wastewater for irrigation. So, the present work aims to assess the impacts of wastewater irrigation on the productivity of three edible weeds (*Cichorium endivia, Sonchus oleraceous* and *Beta vulgaris*) and its effect on the nutritional value of plants and its risk on human health. This study will focus on Shibin Al Kanater region, and the physicochemical characteristics of drainage water, canal water, drainage water-irrigated soils and canal-irrigated soils were estimated. The vegetative and traits of edible weeds were determined including their photosynthetic pigments, organic and inorganic nutrients content, and heavy metals content. The health risk index (HRI) associated with consumption of polluted plants was created using the estimated exposure factor of a crop to the oral reference dosage of the toxic metal. The main results showed that biomass productivity of *S. oleraceous, B. vulgaris* and* C. endivia* increased due to drainage water irrigation with increasing percentage as 27.9, 19.6, and 19.1%, respectively. Irrigation with drainage water significantly increased the photosynthetic pigments of edible weeds. Irrigation with drainage water increased carbohydrate content, crude protein, total soluble sugar, and gross energy in all studied weeds. *C. endivia*, *S. oleraceus* and* B. vulgaris* plants irrigated with canal and drainage water could accumulate Fe, Zn, Cu, and Co in their roots. *C. endivia, S. oleraceus* and *B. vulgaris* plants irrigated with canal water indicated HRI more than the unit for Mn, Cu, Pb, and Cd. This research advises that regulation be put in place to prohibit irrigation using untreated drainage and to restrict the discharge of industrial, domestic, and agricultural wastewater into irrigation canals.

## Introduction

Pollution refers to introducte contaminants into the natural environments and causes adverse change in ecosystem components^1^ and affect soil, plant, ground and surface water, and consequently human health^2,3^. Three factors determine the severity of a pollutant like its chemical nature, concentration, and the persistence^4^. Urban areas have become seriously contaminated zones by heavy metals derived from high density population and intensive industry^5,6^.

Contamination with heavy metals is associated with the rapid development of urbanization, industrialization, manufacturing and mining, that pose increasing threat to human health^7^, and eventually destroying the sustainable development of environmental resources^8^. Transfer heavy metals through the food chains and their accumulation in the human body cause potential health hazards which may leads to death^9^. Water pollution with heavy metals is the most effective factor on all constituents of the agroecosystems and consequently on the food chain^10,11^.

Water pollution with heavy metals is interested issue for a lot of researchers^12^. Many authors interested in studying the effect of the heavy metals on plant crops^13–15^, and others searching about plants have high potency in improving wastewater and contaminated soils^16–18^. By 2025, water scarcity will affect more than half of the world's population, affecting economies, social advancement and person health^19^. Unfortunately, nations suffering from water shortages rely on inadequate alternatives, such using untreated wastewater^20^. In agricultural systems where absence of fresh water in irrigation canals, wastewater can be used due to the accumulation of organic residues and mineral nutrients, but also contains hazardous metals^21^. Rapid increases in people, production, construction, and domestic water supplies have led to rapid increases in wastewater production^22^.

A key global public concern is food safety, in which crop plants (cereals, legumes and vegetables), are abundant in vitamins, fibres, minerals, antioxidants, and important elements; are a common food source for many cultures around the world^23^.

Vegetables are edible plants and storage food in roots, stems, leaves or fruits^24^. Vegetables constitute of essential diet components by contributing protein, vitamins, calcium, iron, and other different nutrients. These elements are the building blocks of human body and aid in the construction of bones, teeth, hair and nails and protects human bodies against the attack of different diseases^25^. Toxic metals accumulation in edible parts of vegetables may cause threat to human health^26^. Weeds that grow among the different crops are known as crop weeds. Many of these weeds can consumed by habitants as fresh or cooked vegetables, these weeds called edible weeds as *Mulva parvilora*, *Sonchus oleracieus*, *Betal vulgaris*, *Cichurium indivia* and others confirmed or not confirmed by FAO^27^.

Treated sewage is a possible supply of water for plants and contains high levels of macro- and micronutrients and heavy metals^28^. Christou et al.^29^, showed that plants extracted heavy metals from contaminated soil through their roots and could translocate them to the edible parts of plant. The problem is further doubled when people consume vegetables irrigated with wastewater without knowing their level of heavy metals.

Bioaccumulation in combination with techniques like phytostabilization and phytodegradation achieves better heavy metal removal^30^. Accumulation of heavy metals and their impacts depends on the ability of the plant species to extract heavy metals from soil, bioaccumates them in roots or translocate them to the vegetative edible parts. According to Singh et al.^31^, vegetables cultivated in contaminated soils accumulate high concentrations of heavy metals.

Many vegetables are known by their potency to accumulate many heavy metals in the edible parts more than the permissible concentrations identifies by FAO/WHO, for examples; *Brassica oleracea* L.^13^, *Malva parviflor*a^16^, *Arachis hypogea*^14^, Spanish, Cabbage and lettuce^32^, *Fragaria ananassa*, *Triticum aestivum*, *Lycopersicon esculentum*, *Saccharum officinarum*, and *Nicotiana tabacum*^33^. Previous studies on all these vegetables recorded reduction in growth triates, biomass and productivity, in addition consuming these contaminated have risk on the human health.

Food insecurity and malnutrition face a lot of people all over the world because of ever-growing human population^34^, especially in the import-dependent countries of Africa^35^. Globally, a 1.02 billion people undernourished are estimated^36^. Wild edible plants are available from their natural habitat and are used as a part of food^37^. Whereas, wild edible plants are a part of cultural and genetic heritage of various zones of the world. During famine, these plants that sources of nutrients and health-promoting constituents have received especial importance in the rural regions^38^.

Balah and Hozyan^39^, recorded forty-four edible weed species during the study in different localities of Egypt. In addition, they recorded high nutritional value of many edible weeds (*Corchorus olitorius*, *Portulaca oleracea*, *Cichorium intybus*, and *Sonchus oleraceus*).

In many growing regions of Egypt, the Nile River does not feed irrigation canals, carrying farmers to use untreated wastewater to water their crops. In addition, edible weeds with its importance receive low attention to the effect of polluted soils irrigated with wastewater on compared to plant crops. Consequently, this study aimed to assess the effect of irrigation with wastewater on the growth, productivity, nutritional value of three natural edible weeds (*Sonchus oleraceous, Beta vulgaris* and *Cichorium endivia*), as well as the potency of the three weeds to accumulate heavy metals and its risk on human health. The significance of this study is to raise awareness of the habitants those consumed these edible weeds and ministries of agriculture, health, and environment about the dangerous using wastewater in irrigating not only crop plants but also associated edible weeds.

## Materials and methods

### Study area

**Figure 1:**
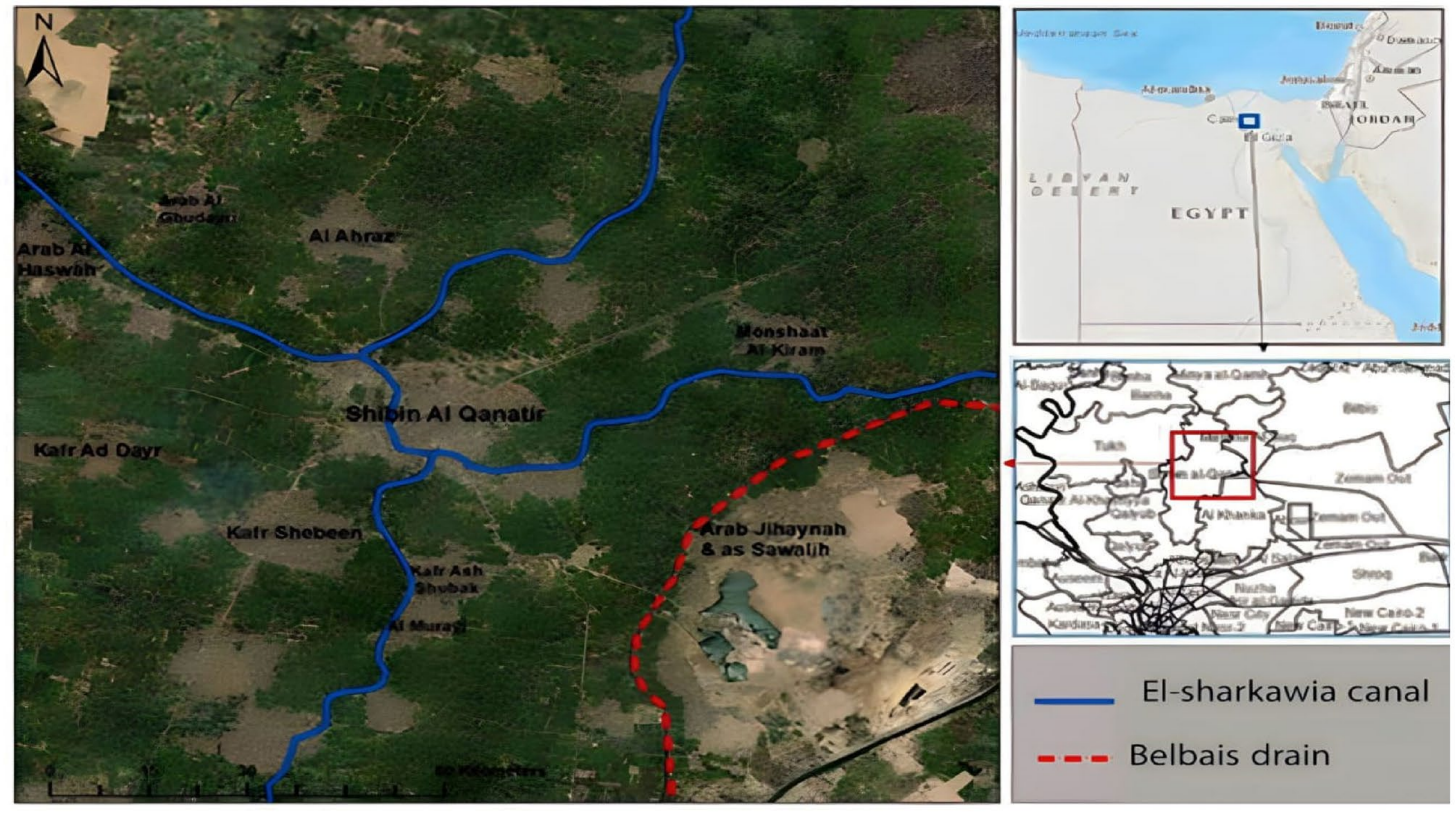
Map location of the study sites.

This study used Shibin Al Kanater area as an illustration of how numerous sectors in Egypt exploited canal and drainage water for crop irrigation. At the southernmost tip of the Nile Delta, in the Al-Qalyubia Governorate, is the Shibin Al Kanater sector. In the research region, there are two locations that are both clean and polluted. The clean sites got their irrigation water from the El-Sharkawia canal, whereas the dirty ones got theirs from the Belbais drain as shown in Fig. 1.

A freshwater irrigation canal called El-Sharkawia originates from the River Nile in the Shoubra El-Kheima district, 12 kms upstream from El-Qanater El-Khairia, where the Nile splits. It travels across the El-Qalyubia Governorate for around 30 kms with an average of 2–4 m depth^22^, 142,000 feddans of surface area, with a width of 5 to 20 m. El-Sharkawia canal is one of the primary sources of drinking water and irrigation in the El-Qalyubia Governorate. It is also regarded as a significant fishing location nearby. While the total irrigation reins are 162,700 feddans, the direct irrigation reins are 1208 feddans. The main Belbais drain empties into the Bahr El-Baqar drain after receiving wastewater from Cairo. Sewage, treated and untreated industrial wastewater are mixed in the drain's water^23^.

### Species of study

Table S1 lists each plant's scientific, and family names with the corresponding photos. Three natural edible weeds; *Cichorium endivia* and *Sonchus oleraceous* are belong to family Asteraceae; and *Beta vulgaris* is belonging to family were selected for this study. The three plants are distributed in agroecosystems and associated to plant crops. Boulos (2000)^40^, and Boulos, (2002)^41^, described the three plants as following:

### *Sonchus oleraceus* L.

Sonchus oleraceus is a plant of Asteraceae Family. The plant is Glabrescent annual with height 10–80 cm, often glandular-hairy above. The stems are simple or branched, angular and hollow. Leaves are variable, lower leaves are simple with narrow winged petiole. Cauline leaves are larger, pinnatifid to pinnatisect, sometimes auriculate, the lobes of variable shape, not or slightly constricted at the base, the terminal lobe much larger than laterals, the margins spinulose-dentate.

### *Cichorium indivia* L.

*Cichorium indivia* is a plant of Asteraceae Family. The plant is glabrous annual, its geight with height 10–80 cm. Its stems are erect and much branched. The plant basal leaves are oblanceolate, pinnatifid to pinnatisect, tapering to a short petiole, the blade margins are denticulate. While the caulme leaves are smaller, entire or denticulate, auriculate.

### *Betavulgaris* L. subsp. *maritima*

*Beta vulgaris* is a plant of Amaranthaceae Family. The plant habit is an annual or short-lived perennial. Its height is ranged between 20 and 80 cm. the stems are erect or decumbent, branched at the stem base and its color is green or reddish. The plant leaves are fleshy and glabrous.

### Water and soil analysis

Water and soil analysis were taken from prefious study of Ahmed et al.^14^. The pH of the soil in the polluted and unpolluted areas ranged from 6.95 (polluted) to 7.01 (unpolluted), and both areas tended to be neutral. In soils watered with drainage water, all anions (Co_3_^2-^, HCO_3_^-^, Cl^-^, and SO_4_^2-^) and cations (Ca^2+^, Mg^2+^, and K^+^) recorded a greater concentration (Table 3). Similar to this, soils irrigated with drainage water showed greater quantities of heavy metals^14^.

### Plant sampling and analysis

In the study sites, five farms that used drainage water and others that used canal water for irrigation were chosen. Appropriate permission was obtained from the farm owners for the study. Five 1.0 × 1.0 m quadrates in each farm were chosen at random. At the end of the growing season, all plants inside each quadrat were collected, wrapped in polyethylene bags, and transported to the lab. To get rid of any debris or dust, the plants were cleaned with tap water, rinsed, and then given another wash with distilled water.

### Growth and yeild parameters

The total number of plants (plants m^−2^) was counted in each square. Root and shoot lengths were measured with a tape measure. The area of a single side leaf was calculated using the tracing paper method. Weed samples were separated into roots and shoots and dried in an oven at 60 °C to a constant dry weight. Each part's constant dry weight was determined. Additionally, the productivity of fresh and dry biomass was determined in kg fed^−1^.

### Photosynthetic pigments

Chlorophyll a, b and carotenoids, were recorded for the studied plants using the spectrophotometeric method recommended and described by Allen^42^.

### Inorganic nutrients

Three mixed samples of each farm's unpolluted and polluted areas were collected for roots as well as shoots of the tested plants. The dried samples were homogenised by milling in a metalless plastic mixer and subsequently used a sieve to pass with a net size of 2 mm. To assess heavy metals and minerals, plant powder (0.2 g) was dissolved in H_2_SO_4_ and HClO_4_ acid mixes to be digested.

The clear digestion product was filtered and diluted to 50 mL twice using deionized H_2_O. Nitrogen content (N) in plant roots and shoots was measured by Kjeldahl method. Using a spectrophotometer (model CECIL CE 1021) and the molybdenum blue technique, the amount of phosphorus was determined. The concentration of magnesium, calcium, and heavy metals including Fe, Co, Cu, Mn, Zn, Pb, and Cd were determined using an atomic absorption spectrophotometer (Shimadzu AA-6200) and the methods described according by Allen^42^.

### Organic nutrients

The nitrogen concentration was multiplied by 6.25 to determine the amount of crude protein (CP)^43^. Total carbohydrates (nitrogen-free extract, “NFE”) and total soluble sugars were extracted as designated by Kennedy and Chaplin^44^, both amounts were estimated colorimetrically using the phenol–sulfuric acid method described by Dubois et al.^45^, using a spectrophotometer with a wavelength of 490 nm. Ether extract (crude fat) content was assessed by extracting the dried matter of the plant samples with ether. The Soxhlet extraction method was used to calculate the crude fibre content (CF)^46^. Digestible crude protein (DCP) was determined via Eq. (1)^47^:

1 $${\text{DCP}}\left( {{\text{as}}\;\% {\text{DM}}} \right) = 0.929\;{\text{CP}}\;\left( {{\text{in}}\;\% {\text{DM}}} \right) - 3.52$$

Total digestible nutrients (TDN) were determined via Eq. (2)^48^:

2 $${\text{TDN}} ({\text{in}} {\% } {\text{dry matter}}) = {0.623} {(100 + 1.25}{\text{ EE}}) - {\text{CP 0.72}},$$

where EE and CP are the ether extract and crude protein percentages, respectively.

The digestible energy (DE) was calculated from Eq. (3)^49^:

3 $${\text{DE }}\left( {{\text{Mcal kg}}^{{ - {1}}} } \right) = 0.0{5}0{\text{4 CP }}\left( \% \right) \, + \, 0.0{\text{77 EE }}\left( \% \right) \, + \, 0.0{\text{2 CF }}\left( \% \right) \, + \, 0.000{377 }\left( {{\text{NFE}}} \right)^{{2}} \left( \% \right) \, + \, 0.0{\text{11 NFE }}\left( \% \right){-}0.{152}.$$

The metabolized energy (ME) was estimated via Eq. (4)^50^:

4 $${\text{ME }} = \, 0.{\text{82 DE}}.$$

The gross energy (GE) was calculated via the Eq. (5)^49^:

5 $${\text{GE }}\left( {{\text{Kcal 1}}00{\text{ g}}^{{ - {1}}} } \right) \, = { 5}.{\text{72 CP }}\left( \% \right) \, + { 9}.{\text{5 EE }}\left( \% \right) \, + { 4}.{\text{79 CF }}\left( \% \right) \, + { 4}.0{\text{3 NFE }}\left( \% \right)$$

### Data analysis

#### Water quality index

Water Quality Index (WQI) is defined as an assessment technique that indicates the combined impact of individual water quality parameters on overall water quality^51^. WQI assesses the adequacy of water quality in the El Sharkweir Canal and Berbais Drainage using a weighted arithmetic water quality index method that classifies water quality based on purity using the most commonly measured water quality variables. The WQI calculation method was adopted by Brown et al.^52^, and the formula for the WQI method is:

$$WQI\, = \,\sum\limits_{i = 1}^{n} {Q_{i} W_{i} } /\sum\limits_{i = 1}^{n} {W_{i} } ,$$

where Q_i_ is the sub quality index of ith parameter (or Q_i_ is the quality rating scale of each parameter). W = weight unit of each parameter, n = number of parameters.

WQI has been classified into 5 classes, the water quality is rated excellent, good, poor, very poor and unfit when the value of the index lies between 0–25, 26–50, 51–75, 76–100 and > 100, respectively Table 1.

**Table 1:** Water quality rating as per weight arithmetic water quality index method.

WQI value	Rating of water quality	Grading
0–25	Excellent	A
26–50	Good	B
51–75	Poor	C
76–100	Very poor	D
Above 100	Unsuitable for irrigation	E

### Bioaccumulation factor (BF)

Concentrations of heavy metals in soil and plants were calculated on a dry weight basis (mg kg^-1^ dry weight). The bioaccumulation factor (BF) is a measure of the plant's ability to concentrate metals in parallel with the concentration of metals in soil^53^, and was calculated as:

$${\text{BF }} = {\text{ C}}_{{{\text{Root}}}} /{\text{C}}_{{{\text{Soil}}}} ,$$

where C_Root_ and C_Soil_ are the heavy metal concentration in plant root and soil, respectively.

### Translocation factor (TF)

The translocation factor or mobilization ratio, are used to determine the relative transfer of metals from underground roots to above-ground shoots of plant species^54^, was calculated as:

$${\text{TF }} = {\text{ C}}_{{{\text{shoot}}}} /{\text{C}}_{{{\text{root}}}} ,$$

where C_shoot_ and C_root_ is the heavy metal concentration in plant shoot and root, respectively.

### Health risk index (HRI)

It is required to determine the level of exposure by identifying the pathways of exposure to the target organisms in order to evaluate the health risk of any pollutants in the edible portions of the plant. The average intake of polluted plants for adults and kids was used to calculate the daily intake of metals (DIM). It was estimated as the following^55^:

$${\text{DIM}} = \,\left( {C_{{{\text{metal}}}} \times C_{{{\text{factor}}}} \times D_{{{\text{food}}\,{\text{intake}}}} } \right)/B_{{{\text{average}}\,{\text{weight}}}} ,$$

where C_metal_ represents heavy metal concentrations in plant part (mg kg^−1^), C_factor_ is a conversion factor, D_food intake_ is daily intake of vegetables, and B_average weight_ is average body weight.

Vegetable dry weights were converted to fresh weights using a conversion factor (0.085)^56^. According to Arora et al.^57^, and Asgari and Cornelis^58^, the average daily consumption for adults and children was 0.345 kg and 0.232 kg, respectively, while the average body weight for adults and children was 55.9 kg and 32.7 kg, respectively.

The health risk index (HRI) associated with consumption of the polluted plants was created using the estimated exposure factor of a crop to the oral reference dosage of the toxic metal^31^. The USEPA states that an HRI value greater than one is hazardous to human health and could harm consumer health. The following are the metals' oral reference doses: 0.001 for Pb and Cd, 3.01 for Co, 0.02 for Ni, 1.5 for Cr, 0.3 for Zn, 0.14 for Mn, 15.0 for Fe, and 0.04 for Cu^59,60^.

### Statistical analysis

The statistical variations between the contaminated and unpolluted locations' plant factors were assessed using an F-test. A one-way analysis of variance (ANOVA-1) was performed using SPSS software^61^, to determine the significance of changes assessed using Duncan's multiple ranges at P 0.05 after the data had been checked for normality.

### Ethics approval and consent to participate

Experimental research, field studies on plants (either cultivated or wild), and all methods were performed in accordance with the relevant institutional, national, and international guidelines and legislation.

## Results

### Water quality index (WQI)

The results showd that the value of WQI of El-Sharkwia canal and Belbais drain were 29.42 and 129.53, respectively with respect to irrigation water. Table 2 indicates that the water quality of El-Sharkwia canal could be classified as good where Belbais drain classified as unsuitable for irrigation.

**Table 2:** WQI and its categorization of El-Sharkwia canal and Belbais drain water for irrigation utilizations.

Source of water	WQI	Category
El-Sharkwia canal	29.42	Good
Belbais drain	129.53	Unsuitable for irrigation

### Vegetative and yield traits of three plants irrigated with canal and drainage water

The impact of irrigation with canal and drainage water on the vegetative and yield features of *C. endivia* plants was shown in Table 3. The findings demonstrated that drainage water irrigation significantly increased plant density (from 4.50 ± 2.12 to 8.00 ± 1.41 Indv./m^2^), shoot length (from 35.34 ± 2.18 to 44.00 ± 0.47 cm) and leaf area (from 105.26 ± 10.50 to 135.0 ± 5.91 cm^2^).

**Table 3:** Vegetative and yield traits of canal and drainage water irrigated edible weeds.

Irrigation water	Plant density (Indv./m^2^)	Plant length (cm)		Shoot fresh weight (g m^−2^)	Shoot dry weight (g m^−2^)	No. of branch/Plant	Leaf area (cm^2^)	fresh biomass productivity (kg f^−1^)	Dry biomass productivity (kg f^−1^)
		Root	Shoot						
*Cichorium endivia*									
Canal water	4.50 ± 2.12	13.83 ± 1.48	35.34 ± 2.18	57.35 ± 1.41	7.11 ± 0.45	13.83 ± 1.70	105.26 ± 10.50	240.9 ± 10.5	29.8 ± 1.42
Drainage water	8.00 ± 1.41	9.84 ± 0.48	44.00 ± 0.47	67.33 ± 2.84	8.44 ± 0.55	12.67 ± 2.01	135.0 ± 5.91	282.8 ± 5.62	35.5 ± 3. 40
F-value	15.77***	7.80*	15.55**	19.82**	11.35**	0.04	18.54*	121.00**	1179.00***
*Sonchus oleraceus*									
Canal water	1.50 ± 0.71	11.25 ± 2.47	35.84 ± 3.62	43.26 ± 2.28	4.68 ± 0.40	7.42 ± 1.29	142.0 ± 2.83	181.7 ± 5.52	19.7 ± 1.40
Drainage water	3.50 ± 1.01	18.00 ± 2.07	43.50 ± 4.92	52.34 ± 1.26	6.00 ± 0.75	16.0 ± 2.07	243.67 ± 8.24	219.8 ± 8.50	25.200 ± 2.80
F-value	11.80**	41.62**	14.24**	24.36*	9.52*	22.85**	51.11***	16.20**	42.00***
*Beta vulgaris*									
Canal water	2.50 ± 0.71	15.17 ± 0.23	35.50 ± 0.71	82.04 ± 2.26	13.60 ± 1.32	6.06 ± 0.39	105.26 ± 10.50	344.57 ± 15.0	57.12 ± 4.2
Drainage water	1.50 ± 0.39	16.75 ± 0.35	40.50 ± 2.58	98.93 ± 1.97	16.26 ± 1.97	6.75 ± 1.77	135.0 ± 5.91	415.5 ± 18.6	68.29 ± 5.1
F-value	2.00	27.99*	14.07*	63.51*	12.53*	0.30	41.54**	98.00**	12.00*

*, **, *** Significant difference at *P* < 0.05, 0.01, 0.001.

Irrigation with drainage water significantly increased shoot fresh and dry weights (from 57.35 ± 1.41 to 67.33 ± 2.84 and from 7.11 ± 0.45 to 8.44 ± 1.55 g m^−2^, respectively), and also increase fresh biomass productivity and dry biomass productivity (from 240.9 ± 10.50 to 282.8 ± 5.62 and from 29.8 ± 1.42 to 35.5 ± 3.40 kg f^−1^), respectively. Compared to irrigation with canal water, irrigation with drainage water significantly decreased the root length of *C. endivia* plants with reduction percentage 28.85%. On the other hand No. of branches/plant nonsignificantly decreased.

Statistical analysis (ANOVA) detected significant variation in vegetative and yield traits between *S. oleraceus* plants irrigated with canal and drainage water. Irrigation with drainage water significantly increased all vegetative and yield traits (Table 3). Plants irrigated with drainage water recorded plant density (3.5 ± 1.01 individual/m^2^), while plants irrigated with canal water recorded plant density (1.50 ± 0.71 individual/m^2^).

In addition, root length, shoot length, shoot fresh and dry weight and no. of branches/plant were increased by 60.0, 21.37, 20.71, 28.2 and 115.6%, respectively. It's noticeable that leaf area of plants irrigated with drainage water significantly increased by 71.6% than plants irrigated with canal water. Drainage water irrigation led to increase fresh and consequently dry biomass productivity with increasing percentage 21.0 and 27.9%, respectively compared to irrigation with canal water.

The statistical analysis of the growth and yield attributes of *B. vulgaris* plants indicated significant variations at *P* < *0.05* and *0.01* between *B. vulgaris* canal and drainage water-irrigated plants. Plant density was higher in farms irrigated with canal water (2.50 ± 0.71 individual/m^2^) than those irrigated with drainage water (1.50 ± 0.39 individual/m^2^).

Table 3 showed that all vegetative and yield traits of *B. vulgaris* plants irrigated with drainage water were significantly higher than the plants irrigated with canal water. Drainage water resulted in increasing shoot fresh weight (from 82.04 ± 2.26 to 98.93 ± 1.97 gm^−2^) and shoot dry weight (from 13.60 ± 1.32 to 16.26 ± 1.97 gm^−2^). Consequently, fresh biomass productivity and dry biomass productivity of *B. vulgaris* plants irrigated with drainage water increased with increasing percentage 20.50% and 19.55% compared to plants irrigated with canal water.

### Photosynthetic pigments of edible weeds

Analysis of the data showed that there was a significant variation at *P* < *0.01* in photosynthetic pigments of *C. endivia* leaves canal and drainage water irrigated. Drainage water irrigation significantly increased Chlorophyll *b* from 1.01 ± 0.12 to 1.22 ± 0.25 mgg^−1^ fr. wt. Carotenoids, on the other hand, dropped from 0.280.015 mgg^−1^ fr.wt. in the leaves of canal water water irrigated plants to 0.170.021 mgg^−1^ fr.wt. in drainage water irrigated plants.

Table 4 showed significant variation in the photosynthetic pigments between *S. oleraceus* canal and drainage water irrigated plants. Chlorophyll *a,* chlorophyll *b* and carotenoids increased in *S. oleraceus* leaves irrigated with drainage water from 1.08 ± 0.31 to 1.19 ± 0.23; 0.87 ± 0.04 to 1.10 ± 0.08 and 0.21 ± 0.02 to 0.22 ± 0.03 mgg^−1^ fresh wt., respectively.

**Table 4:** Photosynthetic pigments (mgg^−1^) of canal and drainage water irrigated edible weeds.

Irrigation water	Photosynthetic pigments (mgg^−1^ fr. wt.)		
	Chlorophyll *a*	Chlorophyll* b*	Carotenoids
*Cichorium endivia*			
Canal water	1.19 ± 0.13	1.01 ± 0.12	0.28 ± 0.015
Drainage water	1.34 ± 0.46	1.22 ± 0.25	0.17 ± 0.021
F-value	0.29	12.84**	8.13**
*Sonchus oleraceus*			
Canal water	1.08 ± 0.31	0.87 ± 0.04	0.21 ± 0.02
Drainage water	1.19 ± 0.23	1.10 ± 0.08	0.22 ± 0.03
F-value	2.16	11.47**	0.20
*Beta vulgaris*			
Canal water	0.62 ± 0.02	0.33 ± 0.04	0.16 ± 0.01
Drainage water	0.76 ± 0.05	0.46 ± 0.06	0.19 ± 0.015
F-value	13.82*	10.75*	8.68*

*, ** Significant difference at *P* < *0.05, 0.01.*

As showed in Table 4 the irrigation with drainage water significantly increased the photosynthetic pigments of *B. vulgaris* plants. Chlorophyll *a* increased from 0.62 ± 0.02 to 0.76 ± 0.05 mgg^−1^, chlorophyll *b* increased from 0.33 ± 0.04 to 0.46 ± 0.06 mgg^−1^ and carotenoids increased from 0.16 ± 0.01 to 0.19 ± 0.015 mgg^−1^ in plant leaves irrigated with drainage water.

### Inorganic nutrients content of edible weeds

The statistical analysis of inorganic nutrients content of *C. endivia* plants recorded significant variations at *P* < *0.05, 0.01* and *0.001* between *C. endivia* plants irrigated with canal and *C. endivia* plants irrigated with drainage water. Irrigation with drainage water significantly increased Ca, N and K content in roots and shoots of *C. endivia* plants. While P content decreased in roots and shoots of *C. endivia* plants irrigated with drainage water than the plants irrigated with canal water from 0.17 ± 0.04 and 0.14 ± 0.02 mgg^−1^ to 0.15 ± 0.02 and 0.13 ± 0.01 mgg^−1^, respectively. In contrast, P and Mg were non-significantly increased in *C. endivia* roots and shoots of plants irrigated with drainage water.

Table 5 showed that irrigation with drainage water significantly decreased Ca content in roots (27.0 ± 2.08 mg g^−1^) and shoots (31.65 ± 2.20 mg g^−1^) of *S. oleraceus* plants than its content in roots (45.94 ± 4.12 mg g^−1^) and shoots (43.50 ± 3.42 mg g^−1^) of plants irrigated with canal water. The content of Mg and P were non-significantly decreased in roots and shoots of *S. oleraceus* plant irrigated with drainage water. On the other hand, irrigation with drainage water resulted in significant increase in nitrogen and potassium contents of *S. oleraceus* roots and shoots than that of plants irrigated with canal water.

**Table 5:** Inorganic nutrients content (mgg^−1^) of canal and drainage water irrigated edible weeds.

Irrigation water	Inorganic nutrients (mgg^−1^ dry wt.)									
	Ca		Mg		N		P		K	
	Root	Shoot	Root	Shoot	Root	Shoot	Root	Shoot	Root	Shoot
*Cichorium endivia*										
Canal water	30.82 ± 1.54	34.60 ± 1.22	5.48 ± 0.36	5.62 ± 0.34	5.48 ± 0.36	5.62 ± 0.34	0.17 ± 0.04	0.14 ± 0.02	1.05 ± 0.01	1.17 ± 0.30
Drainage water	38.93 ± 2.05	38.30 ± 1.44	5.83 ± 0.42	6.03 ± 0.52	5.83 ± 0.42	6.03 ± 0.52	0.15 ± 0.02	0.13 ± 0.01	2.54 ± 0.07	2.19 ± 0.05
F-value	37.65***	15.43*	1.28	2.04	1.28	2.04	0.64	0.45	1.29**	1.87*
*Sonchus oleraceus*										
Canal water	45.94 ± 4.12	43.50 ± 3.42	6.05 ± 0.52	6.13 ± 1.05	3.42 ± 0.20	4.50 ± 0.26	0.18 ± 0.02	0.22 ± 0.02	2.29 ± 0.56	2.86 ± 0.81
Drainage water	27.00 ± 2.08	31.65 ± 2.20	5.81 ± 0.49	6.33 ± 0.67	5.68 ± 0.32	5.25 ± 0.18	0.15 ± 0.02	0.20 ± 0.03	3.10 ± 0.94	3.44 ± 1.51
F-value	28.64**	46.53***	2.15	0.88	16.4**	8.45*	0.72	0.56	5.47**	7.39*
*Beta vulgaris*										
Canal water	32.46 ± 2.14	31.35 ± 2.45	6.64 ± 0.68	7.87 ± 0.97	0.15 ± 0.01	0.16 ± 0.01	2.04 ± 0.10	2.29 ± 0.12	1.13 ± 0.05	1.97 ± 0.11
Drainage water	27.01 ± 1.22	30.37 ± 3.14	6.55 ± 0.84	7.53 ± 1.04	0.21 ± 0.02	0.22 ± 0.01	2.95 ± 0.19	3.15 ± 0.25	1.42 ± 0.30	2.86 ± 0.81
F-value	10.52*	1.25	0.84	0.69	8.26*	11.62**	7.58	9.42*	4.27	8.17*

*, **, *** significant difference at *P* < 0.05, 0.01, 0.001.

The statistical analysis indicated significant variation in calcium, phosphorus and nitrogen content between *B. vulgaris* plants irrigated with canal and drainage water. Table 5 showed that the highest root and shoot contents of Ca (32.46 ± 2.14 and 31.35 ± 2.45 mgg^−1^) were recorded in *B. vulgaris* plants irrigated with canal water than in plants irrigated with drainage (27.01 ± 1.22 and 30.37 ± 3.14 mgg^−1^) water. On the other hand, the highest root and shoot contents of P (0.21 ± 0.02 and 0.22 ± 0.01 mgg^−1^) were recorded in plants irrigated with drainage water. Also, N and K significantly increased in shoots of plants irrigated with drainage water (3.15 ± 0.25 and 2.86 ± 0.81 mgg^−1^) than those irrigated with canal water (2.29 ± 0.12 and 1.97 ± 0.11 mgg^−1^).

### Organic nutrients content of edible weeds

Table 6 showed significant variation in total soluble sugar, crude protein and crude fibers between *C. endivia* plant leaves irrigated with canal water and plant leaves irrigated with drainage water. Total carbohydrates (NFE), total soluble sugars (TSS), crude protein (CP) and crude fats (EE) increased in plants irrigated with drainage water than those irrigated with canal water from 12.03 ± 0.64, 3.2 ± 0.11, 19.3 ± 1.24 and 2.0 ± 0.08% to 13.33 ± 0.72, 4.5 ± 0.14, 30.0 ± 1.85 and 2.1 ± 0.12%, respectively. On the other hand, irrigation with drainage water had negative effect on the content of crude fibers (CF).

**Table 6:** Organic nutrients content of canal and drainage water irrigated edible weeds.

Irrigation water	Organic nutrients content (%)				
	NFE	TSS	CP	EE	CF
*Cichorium endivia*					
Canal water	12.03 ± 0.64	3.2 ± 0.11	19.3 ± 1..24	2.0 ± 0.08	15.34 ± 0.88
Drainage water	13.33 ± 0.72	4.5 ± 0.14	30.0 ± 1.85	2.1 ± 0.12	12.83 ± 0.72
F-value	1.20	7.85*	48.68***	1.24	18.44*
*Sonchus oleraceus*					
Canal water	36.33 ± 0.58	8.20 ± 0.71	28.13 ± 1.45	3.90 ± 0.25	14.61 ± 1.23
Drainage water	40.92 ± 1.24	10.70 ± 0.75	32.81 ± 2.14	5.60 ± 0.44	12.91 ± 0.88
F-value	25.62**	8.66*	9.18*	11.24**	9.65*
*Beta vulgaris*					
Canal water	12.55 ± 0.85	2.70 ± 0.20	14.31 ± 1.37	3.80 ± 0.32	15.10 ± 1.45
Drainage water	15.57 ± 1.26	3.50 ± 0.37	19.69 ± 1.68	1.10 ± 0.08	9.55 ± 0.79
F-value	9.65*	8.42*	15.24**	12.45**	23.45**

*, **, *** significant difference at *P* < 0.05, 0.01, 0.001.

The data in Table 6 showed that irrigation with drainage water significantly increased total carbohydrates (NFE), total soluble sugars (TSS), crude protein (CP) and crude fats (EE) of *S. oleraceus* shoots to 40.92 ± 1.24, 10.70 ± 0.75, 32.81 ± 2.14 and 5.60 ± 0.44%, respectively from 36.33 ± 0.58, 8.20 ± 0.71, 28.13 ± 1.45 and 3.90 ± 0.25%, respectively in plant shoots irrigated with canal water. On the other hand, irrigation with drainage water significantly decreased the content of crude fibers.

The data in Table 6 showed significant variation in total carbohydrates (NFE), Total soluble sugar (TSS), crude protein (CP), crude fats (EE) and crude fibers (CF) content between *B. vulgaris* plants irrigated with canal and *B. vulgaris* plants irrigated with drainage water. Drainage water significantly increased NFE, TSS and CP content in the plant shoots (15.57 ± 1.26, 3.50 ± 0.37 and 19.69 ± 1.68%) than in plant shoots irrigated with canal water (12.55 ± 0.85, 2.70 ± 0.20 and 14.31 ± 1.37%, respectively). On the other side, irrigation with canal water significantly increased EE (3.80 ± 0.32%) and CF (15.10 ± 1.45%) content than those irrigated with drainage water (1.10 ± 0.08, 9.55 ± 0.79%, respectively).

### Nutritional value of edible plants

Statistical analysis detected significant variation in nutritive value elements of *C. endivia* plants irrigated with canal and drainage water (Table 7). Irrigation with drainage water increased the digestible crude protein (DCP) from 14.4 ± 0.75 to 24.4 ± 1.04% but decreased the total digestible nutrients (TDN) from 49.9 ± 2.41 to 42.3 ± 2.06%. Digestible energy (DE), metabolized energy (ME) and gross energy (GE) were increased due to irrigation with drainage water.

**Table 7:** Nutritive value of canal and drainage water irrigated edible weeds shoots.

Irrigation water	Nutritive value				
	DCP	TDN	DE	ME	GE
	%		(Kcal kg^−1^)		
*Cichorium endivia*					
Canal water	14.4 ± 0.75	49.9 ± 2.41	1.5 ± 0.06	0.7 ± 0.01	251.0 ± 16.22
Drainage water	24.4 ± 1.04	42.3 ± 2.06	2.0 ± 0.07	1.0 ± 0.02	306.7 ± 20.4
F-value	45.8***	22.8**	6.25*	9.27*	282.34***
*Sonchus oleraceus*					
Canal water	22.6 ± 0.65	45.1 ± 1.74	2.8 ± 0.08	1.4 ± 0.11	414.2 ± 3.75
Drainage water	27.0 ± 0.75	43.0 ± 1.04	3.3 ± 0.04	1.6 ± 0.12	467.4 ± 5.62
F-value	33.4**	2.56	8.66*	0.54	182.32***
*Beta vulgaris*					
Canal water	9.8 ± 0.62	55.0 ± 1.45	1.4 ± 0.11	0.7 ± 0.06	253.1 ± 6.28
Drainage water	14.8 ± 0.87	49.0 ± 1.33	1.3 ± 0.10	0.7 ± 0.04	219.9 ± 4.54
F-value	20.32***	22.5**	0.84	0.01	168.54***

*, **, *** significant difference at *P* < 0.05, 0.01, 0.001.

As shown in Table 7, *S. oleraceus* plants irrigated with drainage water significantly increased the digestible crude protein (DCP), digestible energy (DE) and gross energy (GE) with increasing percentage 19.46, 17.86 and 12.84%, respectively. On the other side, the total digestible nutrients (TDN) and metabolized energy (ME) showed a non significant variation between plants irrigated with canal and plants irrigated with drainage water.

It was observed in Table 7, irrigation with drainage water significantly increased the DCP from 9.8 ± 0.62 to 14.8 ± 0.87%. On the other hand, TDN and GE were significantly decreased in plants irrigated with drainage water. Digestible and metabolized energy didn't showed significant difference between plant seeds irrigated with canal and drainage water.

### Heavy metals content of edible weeds

Table 8 showed significant variation in heavy metals content between *C. endivia* plants (roots and shoots) irrigated with canal and drainage water. Iron recorded higher significant concentration (421.6 ± 22.4 mgkg^−1^) in the plant roots irrigated with drainage water than the plant roots irrigated with canal water (246.1 ± 16.5 mgkg^−1^). On the other hand, the plant shoots irrigated with canal water had higher significant concentration of Fe than those irrigated with drainage water. Other heavy metals, Mn, Zn, Cu, Co, Pb and Cd significantly increased in roots and shoots of *C. endivia* plants irrigated with drainage water.

**Table 8:** Heavy metals content of canal and drainage water irrigated edible weeds.

Heavy metals	Heavy metals content (mg kg^−1^)					
	Roots irrigated with canal water	Roots irrigated with drainage water	F-value	Shoots irrigated with canal water	Shoots irrigated with drainage water	F-value
*Cichorium endivia*						
Fe	246 ± 16.5	421 ± 22.4	16.25**	209 ± 5.7	164 ± 10.4	9.33*
Mn	100 ± 9.2	227 ± 14.9	34.58***	144 ± 10.2	167 ± 10.8	26.42**
Zn	262 ± 18.7	448 ± 20.8	28.65**	276 ± 6.9	295 ± 17.4	17.54*
Cu	50 ± 3.4	110 ± 8.9	42.58***	20 ± 3.1	80 ± 7.1	285.36***
Co	1 ± 0.24	1.32 ± 0.28	0.15	2 ± 0.45	2.56 ± 0.37	0.14
Pb	2.14 ± 0.34	4.3 ± 0.98	1.98**	3.08 ± 0.51	8.21 ± 2.7	5.45***
Cd	0.24 ± 0.01	1.55 ± 0.45	0.87*	0.62 ± 0.02	2.78 ± 0.31	1.38***
*Sonchus oleraceus*						
Fe	256 ± 21.8	183.14 ± 10.74	114.22**	189 ± 21.7	174.8 ± 11.8	11.54*
Mn	125 ± 7.54	178 ± 9.56	24.65*	124 ± 2.54	258 ± 5.72	13.54***
Zn	307 ± 6.78	338 ± 8.59	20.75*	298 ± 5.78	305 ± 10.45	32.17*
Cu	44.5 ± 3.68	93.28 ± 6.42	245.52***	60.64 ± 5.42	182.12 ± 15.42	234.38***
Co	0.62 ± 0.11	2.02 ± 0.28	8.26*	0.32 ± 0.07	1 ± 0.36	18.34**
Pb	5.14 ± 0.18	8.6 ± 0.38	10.22*	3.08 ± 0.24	6.21 ± 0.54	21.17**
Cd	0.34 ± 0.0146	3.75 ± 0.3	30.41**	0.45 ± 0.0346	5.62 ± 0.45	18.56***
*Beta vulgaris*						
Fe	197 ± 15.44	248 ± 16.02	95.47***	147 ± 12.87	175 ± 13.49	18.78*
Mn	121 ± 13.8	178 ± 14.25	168.5***	124 ± 14.2	258 ± 16.52	99.65***
Zn	207 ± 16.2	338 ± 16.55	65.42**	198 ± 15.3	305 ± 12.78	8.64
Cu	46.6 ± 5.7	79.4 ± 6.2	174.2***	40.25 ± 3.9	70.85 ± 5.7	136.4**
Co	0.21 ± 0.01	3.42 ± 0.4	10.28**	0.06 ± 0.0002	2.2 ± 0.1	25.32**
Pb	3.24 ± 0.24	8.32 ± 0.79	26.14**	1.89 ± 0.1	7.08 ± 0.58	32.26**
Cd	0.4 ± 0.001	5.62 ± 0.46	44.18***	0.3 ± 0.002	3.75 ± 0.31	84.33***

*, **, *** Significant difference at *P* < 0.05, 0.01, 0.001.

From data illustrated in Table 8, the statistical analysis showed significant variation for all investigated heavy metals (Fe, Mn, Zn, Cu, Co, Pb and Cd) in *S. oleraceus* plants (roots and shoots) irrigated with canal and drainage water. Iron significantly increased in roots (256.61 ± 21.80 mgkg^−1^ dry wt.) and shoots (189.42 ± 21.70 mgkg^−1^ dry wt.) of *S. oleraceus* plants irrigated with canal water than plants irrigated with drainage water (183.14 ± 10.74 and 174.80 ± 11.80 mgkg^−1^ dry wt. for root and shoot, respectively). On the other hand, Mn, Zn, Cu, Co, Pb and Cd recorded higher significant concentration in roots (178.00 ± 9.56, 338.00 ± 8.59, 93.28 ± 6.42, 2.02 ± 0.28, 8.60 ± 0.38 and 3.75 ± 0.30 mgkg^−1^ dry wt.) and in shoots (258.00 ± 5.72, 305.00 ± 10.45, 182.12 ± 15.42, 1.0 ± 0.36, 6.21 ± 0.54 and 5.62 ± 0.46 mgkg^−1^ dry wt., respectively) of *S. oleraceus* plants irrigated with drainage water than the concentration in roots (125.00 ± 7.54, 307.00 ± 6.78, 44.50 ± 3.68, 0.62 ± 0.11, 5.14 ± 0.18 and 0.34 ± 0.0146 mgkg^−1^ dry wt.) and in shoots (124.00 ± 2.54, 298.00 ± 5.78, 60.64 ± 5.42, 0.32 ± 0.07, 3.08 ± 0.24 and 0.45 ± 0.0346 mgkg^−1^ dry wt., respectively) of *S. oleraceus* plants irrigated with canal water.

Statistically, there was high significant difference in concentrations of all heavy metals (Fe, Mn, Zn, Co, Cu, Pb and Cd) between the plants irrigated with canal and drainage water (Table 8). Roots and shoots of *B. vulgaris* plants irrigated with drainage water recorded higher significant concentration of Fe (248.0 ± 16.02 and 175.0 ± 13.49 mg kg^−1^) than that irrigated with canal water (197.0 ± 15.44 and 147.0 ± 12.87 mg kg^−1^), respectively. The highest significant difference (at P < *0.001)* was recorded for Cu, Mn and Cd between plants irrigated with drainage water and plants irrigated with canal water. It was noted that the concentration of Mn and Zn were the dominant heavy metals in roots (178.00 ± 14.25 and 338.00 ± 16.55 mg kg^−1^) and shoots (258.00 ± 16.52 and 305.00 ± 12.78 mg kg^−1^) of *B. vulgaris* plants irrigated with drainage water.

### Bioaccumulation and translocation factors of heavy metals for edible weeds

#### Bioaccumulation factors

**Figure 2:**
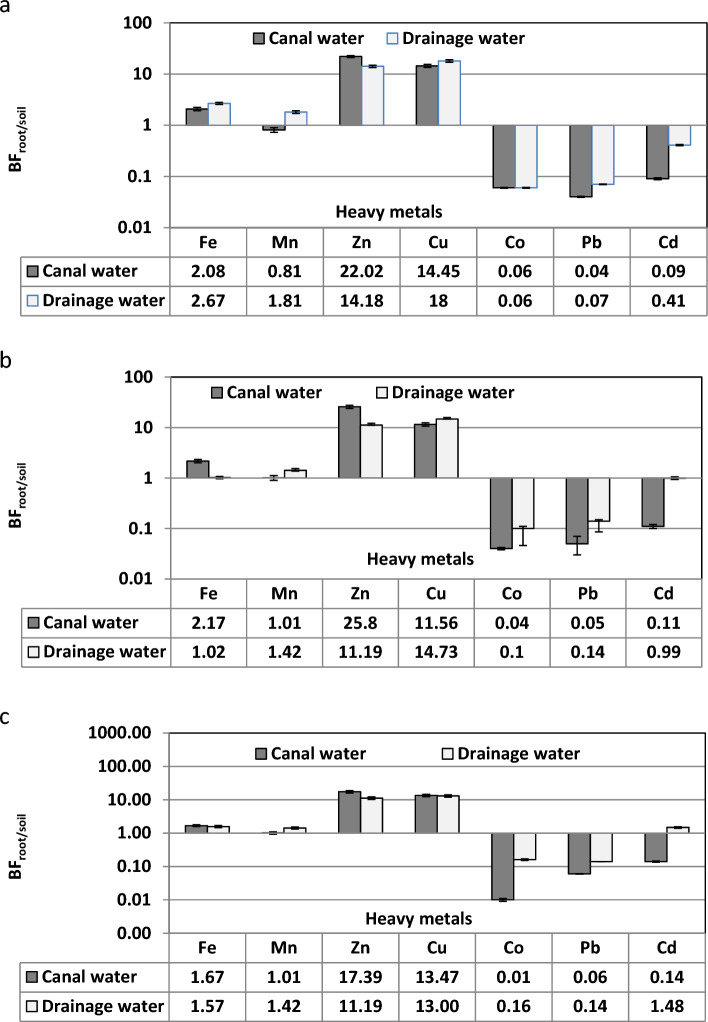
Bioaccumulation factor (BF_root/soil_) of heavy metals by three edible weeds; (**a**) *Cichorium endivia*, (**b**) *Sonchus oleraceus* and (**c**) *Beta vulgaris*.

Bioaccumulation factors of heavy metals by *C. endivia* differed among the investigated heavy metals. Figure 2a indicated that *C. endivia* irrigated with canal and drainage water bioaccumulated Fe, Cu and Zn more than the unit which ranged between 2.08 and 2.67 (For Fe) and 22.02 and 14.83 (for Zn), respectively.

Figure 2b illustrated that bioaccumulation factor (BF) of heavy metals from soil to roots of *S. oleraceus* was less than the unit for Co, Pb and Cd. While the BF of Fe, Mn, Zn and Cu was more than the unit in the plants irrigated with canal and drainage water. Iron, Mn, Zn and Cu recorded the highest BF in *S. oleraceus* plants irrigated with canal water (2.17, 1.01, 25.80 and 11.56) and drainage water (1.02, 1.42, 11.19 and 14.73, respectively). While Co, Pb and Cd recorded BF less than the unit in the plants irrigated with canal and drainage water.

The *Beta vulgaris* plants irrigated with drainage water had high potential to accumulate all heavy metals, except Co and Pb in their roots. The BF of Fe, Mn, Zn, Cu and Cd was greater than the unit Fig. 2c.

#### Translocation factors

**Figure 3:**
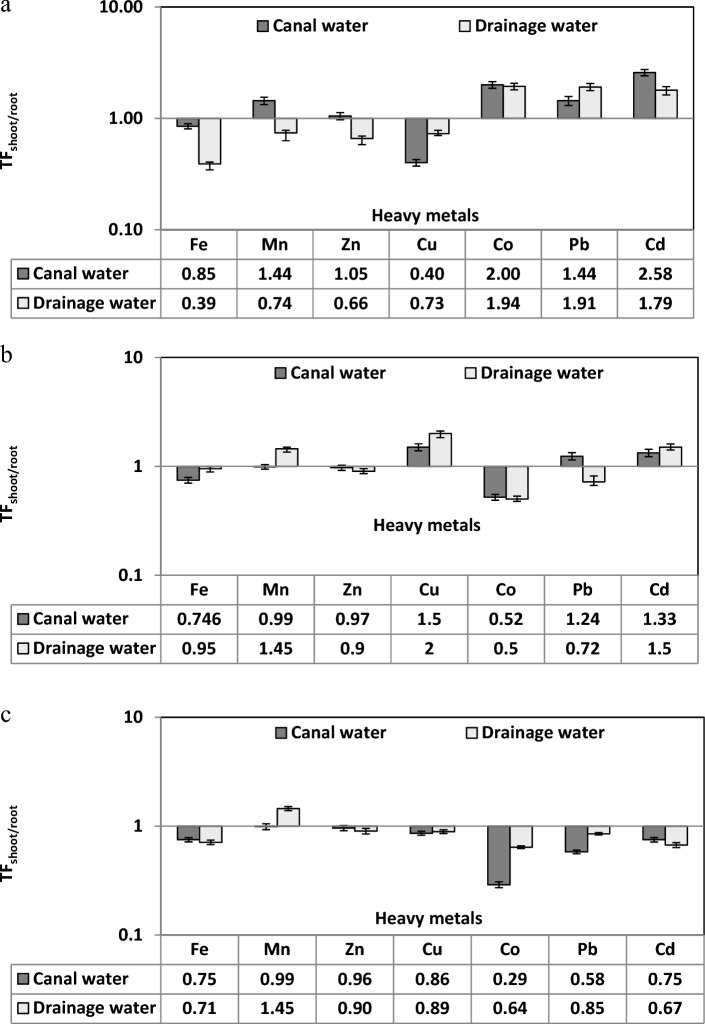
Translocation factor (TF_shoot/root_) of heavy metals by three edible weeds; (**a**) *Cichorium endivia*, (**b**) *Sonchus oleraceus* and (**c**) *Beta vulgaris*.

Translocation factor of the heavy metals Mn (1.44), Zn (1.05), Co (2.00), Pb (1.44) and Cd (2.58) of *C. endivia* plants irrigated with canal water were more than the unit. While the TF for Pb, Co and Cd of the plants irrigated with canal and drainage water were more than unit Fig. 3a. On the other hand, Fig. 3b showed that TF was more than unit for Cu, Pb and Cd in the *S. oleraceus* plants irrigated with canal water (1.50, 1.24 and 1.33) and for Mn, Cu and Cd in the plants irrigated with drainage water (1.45, 2.00 and 1.50). Although, Fe, Zn and Co recorded TF less than the unit in *S. oleraceus* plants irrigated with canal and drainage water. The TF for Mn and Zn in *S. oleraceus* plants irrigated with canal water and for Fe and Zn in plants irrigated with drainage water were considered critical TF as they had TF closest to the unit. The TF from root to shoot (TF_shoot/root_) of Mn had critical value (0.99) in plants irrigated with canal water, and it increased to 1.45 in plants irrigated with drainage water. *Beta vulgaris* plants recorded TF of Fe, Zn, Cu, Co, Pb and Cd lower than the unit in plants both irrigated with canal and drainage water Fig. 3c.

#### Daily intake and health risk index of heavy metals for edible weeds

**Figure 4:**
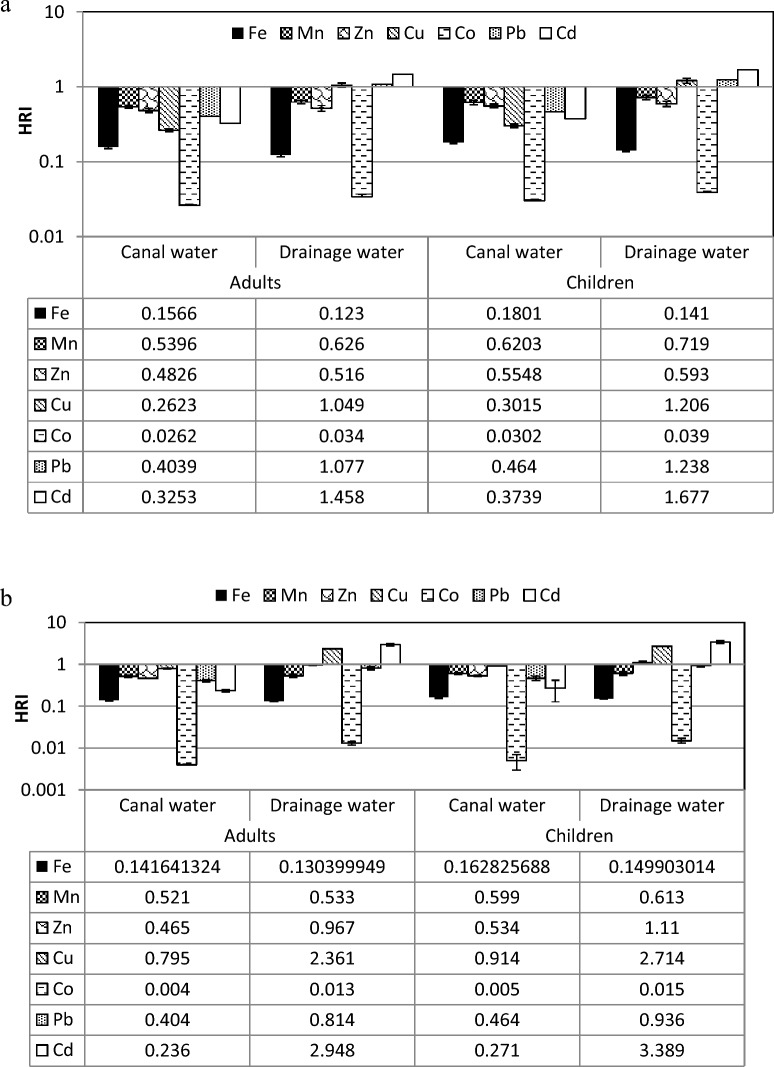

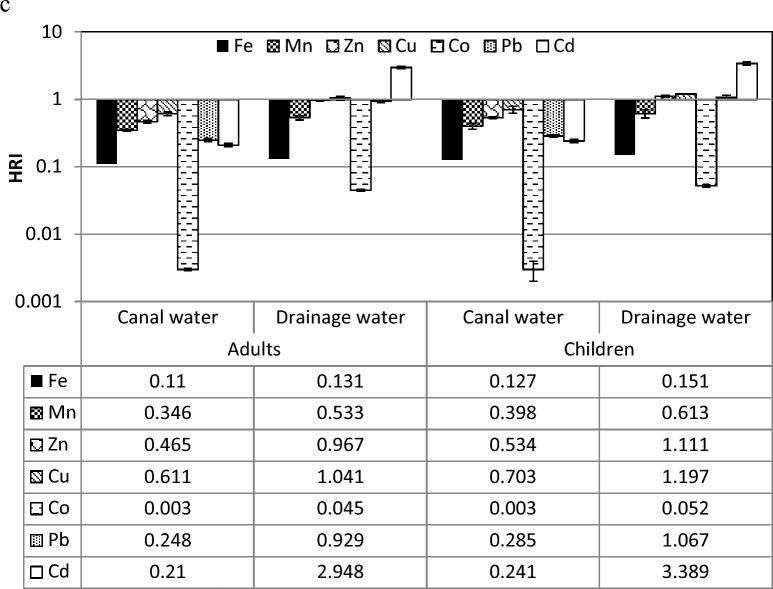
Adults and children health risk index (HRI) via intake of heavy metals in canal and drainage water irrigated edible weeds, where (**a**) *Cichorium endivia,* (**b**) *Sonchus oleraceus*, and (**c**) *Beta vulgaris*.

As shown in Table 9, the daily intake of heavy metals (DIM) in the *C. endivia* leaves irrigated with canal water ranged between 0.000 (for Cd) and 0.145 mg day^−1^ (for Zn) for adults and between 0.000 (for Cd) and 0.166 mg day^−1^ (for Zn) for children. While DIM of the plants irrigated with drainage water ranged between 0.001 (for Co and Cd) and 0.155 mg day^−1^ (for Zn) for adults and between 0.000 (for Cd) and 0.178 mg day^−1^ (for Zn) for children. As shown in Fig. 4a, *C. endivia* plants irrigated with canal water showed health risk index (HRI) less than the unit for adults and children for all heavy metals. On the other side, Cu, Pb and Cd showed HRI more than the unit for plants irrigated with drainage water for adult (1.049, 1.077 and 1.458) and children (1.206, 1.238 and 1.677 mg day^−1^, respectively).

**Table 9:** Adults and children's daily intake of each heavy metal (mg day^−1^) in canal and drainage water irrigated edible weeds for individual.

Heavy metals	Daily intake of metals (mg day^−1^)			
	Adult		Children	
	Canal water	Drainage water	Canal water	Drainage water
*Cichorium endivia*				
Fe	0.110	0.086	0.126	0.099
Mn	0.076	0.088	0.087	0.101
Zn	0.145	0.155	0.166	0.178
Cu	0.010	0.042	0.012	0.048
Co	0.001	0.001	0.001	0.002
Pb	0.002	0.004	0.002	0.005
Cd	0.000	0.001	0.000	0.002
*Sonchus oleraceus*				
Fe	0.0991	0.0913	0.1140	0.1049
Mn	0.0651	0.1353	0.0748	0.1556
Zn	0.1563	0.1600	0.1797	0.1839
Cu	0.0318	0.0944	0.0366	0.1086
Co	0.0002	0.0005	0.0002	0.0006
Pb	0.0016	0.0033	0.0019	0.0037
Cd	0.0002	0.0029	0.0003	0.0034
*Beta vulgaris*				
Fe	0.077	0.092	0.089	0.106
Mn	0.065	0.135	0.075	0.156
Zn	0.104	0.160	0.119	0.184
Cu	0.021	0.037	0.024	0.043
Co	0.0002	0.001	0.0001	0.001
Pb	0.001	0.004	0.001	0.004
Cd	0.0001	0.002	0.000	0.002

Table 9 showed that the daily intake of metals (DIM) for all heavy metals of *S. oleraceus* leaves irrigated with drainage water are more than of those irrigated with canal water for adult and children.

Figure 4b illustrated that all heavy metals recorded health risk index (HRI) less than the unit for *S. oleraceus* plants irrigated with drainage water for adult and children except that of Cd and Cu (2.948 and 2.361) for adults and Mn, Cu and Cd (1.111, 2.714, and 3.389) for children were more than the unit. The Cu in *S. oleraceus* plants that was irrigated with canal water recorded critical HRI for adults (0.795) and children (0.914) as a result of consumption leaves of *S. olerace*us plants.

The daily intake of metals (DIM) was ranged between 0.0001 for Cd and 0.184 mg day^−1^ for Zn when children consumed plants grown in the soils irrigated with drainage water (Table 9). Moreover, Cu and Cd showed high health risk index for adult from consuming *B. vulgaris* plants irrigated with drainage water. The HRI of Cu and Cd for adults increased in plants irrigated with drainage water from 0.611 to 1.041 and from 0.21 to 2.948, respectively. For children, Mn, Cu, Pb and Cd had HRI more than the unit (1.111, 1.197, 1.067, and 3.389) in the plants irrigated with drainage water and for plants irrigated with canal water (0.534, 0.703, 0.285, and 0.241) as presented in Fig. 4c.

## Discussion

Shortages of water is a serious problem for farmers, causes them to use wastewater to irrigate their crops. Wastewater irrigation has a long tradition dating back hundreds of years and performs better in industrialised nations than in developing nations^62^. However, most farmers focus just with increasing agricultural yields and are uninformed of the advantages and environmental dangers of wastewater^63^. Soil provides a direct pathway for plant heavy metal contamination via root uptake. Facilities that use wastewater for irrigation may collect and accumulate heavy metals above the highest allowable level, which has serious implications for public health^64,65^.

Excessive exposure to heavy metals can increase the generation of reactive oxygen species (ROS), which can cause changes in the plant cell cycle, cell division, and even chromosomal abnormalities^66^. In addition, excessive ROS generation results in genotoxicity, lipid peroxidation, and protein oxidation.

Irrigation with drainage water resulted in an increase of all vegetative and yield traits in the three selected edible weeds (*Sonchus oleraceus, Cichorium endivia* and *Beta vulgaris*). These findings are in agreement with Ali et al.^67^, they reported that all growth metrics were significantly higher in waste water irrigated plant species than in fresh water plants for the three selected leafy vegetable plants (Coriander (*Corianderum sativum*), Purslane (*Portulaca oleracae*), and Lactuca (*Lactuca sativa*)). Also, the current findings are in alignment with Faizan et al.^68^ who found that all the growth and yield parameters were found to increase due to wastewater application. This may be attributable to the presence of NH_4_^+^ and NO_3_^-^, the two ionic forms of nitrogen that are important for raising the number of meristematic cells^68^.

The use of wastewater for irrigation may be a rich source of nutrients and other vital components for the growth of plants. In contrast to fresh water, wastewater actually has a higher quantity of organic matter and nutrients. Therefore, the soil's nutrient accumulation will make it simple for plants to obtain these nutrients^67^.

Weeds pigments were significantly increased by irrigating with drainage water, these finding are in agreement with Thapliyal et al.^69^, and Faizan et al.^68^. This rise is attributed to the increase of Mg^2+^ content and other nutrients present in the sewage water. From these nutrients, nitrogen which is indirectly related to one of the basic plant physiological processes, the photosynthesis, as 70% of N in plant leaves exists in chloroplast and most of it is used for the synthesis of the photosynthetic apparatus. Photosynthetic rate, thereby improving most yield attributes including number of branches/plants, fresh and dry biomass productivity.

Studied weeds irrigated with drainage water showed an increase in the content of NPK like the reports of Shah et al.^70^, and Tabassum et al.^71^ for other plants. This may be attributed to excess nutrients in the drainage resulting in greater leaf area development, ultimately extracting more nutrients and water^68^.

Wastewater irrigation has raised nitrogen contribution, which has increased the N content and thus increased protein in plants. The continuous availability of additional vital nutrients through wastewater also aided the growth and productivity of the investigated weeds.

Compared to plant leaves irrigated with canal water, the GE of weeds increased in drainage water-irrigated plant leaves, where GE of *C. endivia* and *S. oleraceus* irrigated with drainage water are 306.7 and 467.4, while in canal water irrigated plants are 251 and 414.2. These results are disagreed with that of Ahmed et al.^14^ on peanut crop, who reported that too much deposit of hazardous metals (Mn, Zn Cu, Co, Pb and Cd,). This may reduce carbohydrates production by breaking the photosynthetic electron transport chain^72^. It should be mentioned that the nature and traits of the plant itself influence the rate at which each attribute develops. Accumulation of heavy metals and their impacts depends on the ability of the plant species to extracte heavy metals from soil, bioaccumates them in roots or translocate them to the vegetative parts.

Edible weeds, *C. endivia* irrigated with canal and drainage water had high ability to absorb and concentrate Fe, Zn, Cu and Co from soil to roots where, their BF more than the unit. While the translocation factor was higher than 1 for Mn, Cu, Co, Pb and Cd in plants irrigated with canal water. Add to that, BF of *S. oleraceus* was more than the unit for Fe, Zn, Cu and Co in the plants irrigated with canal and drainage water, and the TF was more than unit for Zn and Cd the in *S. oleraceus* plants irrigated with canal water and for Zn, Cu and Cd in plants irrigated with drainage water. The *B.vulgaris* plants irrigated with drainage water had high ability to accumulate Fe, Zn, Cu and Co in their roots and translocate Mn, Zn, Cu and Cd to the plant shoots with TF more than the unit in the plants irrigated with canal and drainage water.

The current outcomes are attributed to the results of Eddy and Ekop^73^, who discovered that plants such as *Phyllanthus amarus* (chanca piedra), *Chromolaena odorate* (awolowos weed), *Stachytarpheta indica* (gervao), *Bryophyllum pinnatum* (life leaf), and *Murraya koenigii* (curry leaf) had the ability to absorb lead, zinc, copper, and nickel from polluted soil. In this context, as a result of soil acidification or complexing processes, the root and microbial exudates may increase the solubility and mobilisation of micronutrients. Zn mobility and availability to plants are governed by rhizosphere biological variables and soil characteristics^74^. Plants take up Zn as free ions from the soil solution, which can come from a variety of sources, including the soil solution, soluble organic complexes, and that is in equilibrium with the soil solution and is adsorbed on various minerals^75^.

Likewise, Samadi et al.^76^, demonstrated that *S. oleraceus* has a good capability for absorbing and storing Cd from the mining area's soil. Chaffei et al.^77^, stated that the physiological processes could be dramatically altered by high levels of Cd in plant tissues. Also, the interaction of heavy metals with DNA and proteins can result in oxidising substances that damage plant components^78^.

The HRI was created to assess the risk posed by hazardous ingredients in food^58^. An HRI greater than 1 is deemed unsafe for human health by the USEPA. Plants of Cichorium endivia irrigated with drainage exhibited greater HRI compared to units of Cu, Pb, and Cd. In plants of *S. oleraceus* and *B. vulgaris* that were irrigated with drainage water, Mn, Cu, and Cd also detected HRIs above 1. One of the most significant global issues is heavy metal environmental pollution. A danger to the human body is posed by heavy metals through, kidney and gastrointestinal impairment, nervous system disorders, vascular damage, skin lesions, birth defects and cancer^79^.

Hyper-accumulators actively take up and translocate metals into their aboveground biomass, while tolerant plants typically restrict soil-root and root-shoot transfers, resulting in much less accumulation in their biomass^80^. From the obtained results, the studied weeds tolerate and survive in soil containing high concentration of heavy metals. Besides, these weeds can be used to remove most of heavy metals from contaminated soil. This remediation approach is non-intrusive, environmentally benign, and it removes metal contaminants from contaminated locations^81,82^.

To keep weeds away from food and to benefit from them, biogas production appears to be one of the best alternatives to obtaining energy from weeds. Also, in process of energy production from weeds, all disease agents threatening human health from waste become inactive^74^.

## Conclusion

In the current study, impact of irrigation using drainage water on *Cichorium endivia, Beta vulgaris* and *Sonchus oleraceus* was evaluated. According to water quality index, results indicate that the water quality of El-Sharkawia canal is good for irrigation usage, while Belbais drain classified as unsuitable for irrigation. High significant concentrations of Fe, Zn, Cu, and Cd were accumulated when soils were irrigated with drainage water. The three edible weeds' vegetative and dry biomass were significantly enhanced by irrigation with drainage water. The photosynthetic pigments of edible weeds significantly increased by irrigating with drainage water. Conversely, irrigating weeds with drainage water increased their gross energy, crude protein, total soluble sugar, and carbohydrate content. *Cichorium endivia, Beta vulgaris *and* Sonchus oleraceus* had capacity to extract and accumulate Fe, Mn, Zn, Cu, and Co in their roots than shoots (edible parts). Water of Belbais drain is not appropriate for irrigation of crops, because toxic metals in excess need to be treated and it had detrimental impact on the economy since it wastes time, effort, and money, and also poses a health risk to people. This work determined the effect of irrigation with wastewater on edible weeds, the extent of accumulation of heavy metals in different parts of these plants, and the potential health risks of their consumption on human health. More research should be conducted to study the effect of wastewater on the physiological processes and anatomical changes of plants as a result of irrigation with wastewater. More efforts must also be made to find solutions to treat wastewater to avoid the health risks and economic damage resulting from its use.

## Drawbacks and recommendations

One of the difficulties encountered by the study is the lack of satisfactory availability of analysis equipment. Also, only few farmers allow entry into their farms and taking samples during the working seasons. The laws that prohibit irrigation with wastewater must be activated and enforced. *C. endivia, B. vulgaris* and *S. oleraceus* can be considered as phytoremediators and they can be used for production of bioenergy.

### Supplementary Information

Supplementary Table S1.

## Data Availability

The datasets used and analyzed during the current study are available from the corresponding author on reasonable request**.**
