# Supplementary material for: Assessment of heavy metal accumulation and health risk in three essential edible weeds grown on wastewater irrigated soil
Source: Sci Rep. 2023 Dec 8;13:21768. doi: 10.1038/s41598-023-48763-5 (PMC10709593; doi:10.1038/s41598-023-48763-5)
Supplement: Supplementary file 1 — Supplementary Table S1. [file 41598_2023_48763_MOESM1_ESM.docx]

**Table S1. Scientific, and family names with the corresponding photos**

| **Species** | **Family** | **Plant’s photo** |
| --- | --- | --- |
| *Sonchus oleraceous* | Asteraceae | **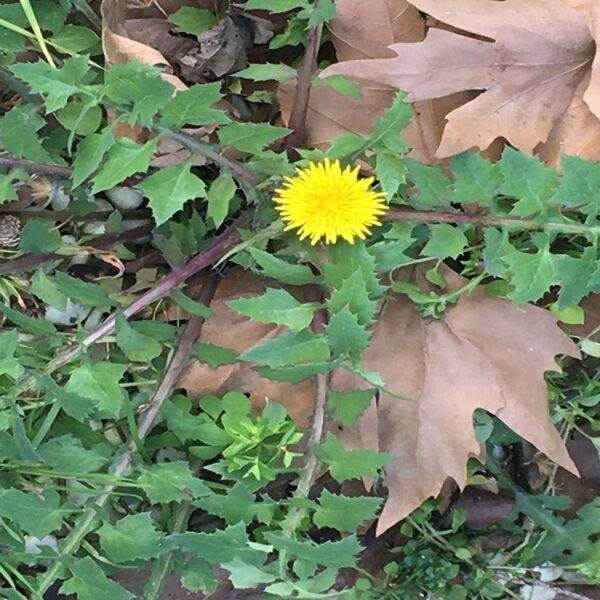** |
| *Beta vulgaris* | [Amaranthaceae](https://en.wikipedia.org/wiki/Amaranthaceae) | **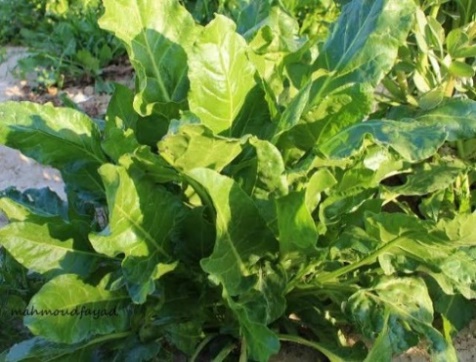** |
| *Cichorium endivia* | Asteraceae | **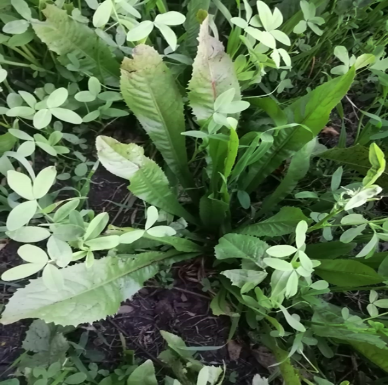** |
